# Navigating challenges and solutions for metal-halide and carbon-based electrodes in perovskite solar cells (NCS-MCEPSC): An environmental approach

**DOI:** 10.1016/j.heliyon.2024.e32843

**Published:** 2024-06-11

**Authors:** Faycal Znidi, Mohamed Morsy, Md. Nizam Uddin

**Affiliations:** Engineering and Physics Department, Texas A&M University, Texarkana, 7101 University Ave, Texarkana, TX, 75503, USA

**Keywords:** M-PSCs, C–PSCs, Efficiency, Low-cost manufacturing, Device architectures, Stability, Durability, Scalability, Environmental impacts, Sustainability, Pb-free materials

## Abstract

The urgent need to shift to renewable energy is highlighted by rising global energy use and environmental issues like global warming from fossil fuel dependency. Perovskite solar cells (PSCs) stand out as a promising option, providing high efficiency and potential for cost-effective production. This study delves into the environmental concerns and viable solutions linked with metal-halide PSCs (M-PSCs) and carbon-based electrode PCSs (C–PSCs). It showcases the swift progress in PSC technology, highlighting its potential to deliver efficient and economical renewable energy options. Yet, the environmental implications of these technologies, especially the utilization of toxic lead (Pb) in M-PSCs and the issues of stability and degradation in C–PSCs, represent considerable hurdles for their broad application and sustainability. The paper details the recent advances in PSCs, focusing on enhancements in device efficiency and stability through innovative material combinations and device designs. Nonetheless, the environmental hazards linked to the dispersal of toxic substances from compromised or deteriorating PSCs into the ecosystem raise significant concerns. In particular, the risk of Pb from M-PSCs contaminating soil and aquatic ecosystems is a pressing issue for human and environmental health, spurring investigations into alternative materials and methods to diminish these impacts. The authors examine several strategies, including the introduction of Pb-free perovskites, encapsulation methods to block the escape of hazardous substances, and the recycling of PSC elements. The study stresses the necessity of aligning technological innovations with considerations for the environment and health, calling for ongoing research into PSC technologies that are sustainable and safe. This review highlights the need for detailed assessments of PSC technologies, focusing on their renewable energy contributions, environmental impacts, and strategies to mitigate these effects. The authors call for a cohesive strategy to develop PSCs that are efficient, cost-effective, eco-friendly, and safe for widespread use.

## 1 Introduction

The escalating global demand for energy and the concomitant environmental repercussions of traditional energy sources underscore the urgent need for sustainable alternatives. Solar photovoltaic (PV) technology, especially PSCs, has rapidly emerged as a frontrunner due to its high efficiency and potential for low-cost production. However, the deployment of PSCs is not without environmental concerns, particularly the use of toxic metal halides in these devices.

Recent advancements in PSC technology have been impressive, with efficiencies increasing from 3.8 % in 2009 to over 25 % in recent years [[1], [2], [3], [4]]. This surge in performance is attributed to innovative material engineering, such as the use of metal-halide and carbon-based electrodes and improvements in device architecture. Despite these technological strides, the environmental impact of PSCs, particularly those containing Pb, poses significant sustainability challenges.

The introduction of C–PSCs represents a promising shift towards more environmentally benign materials in the PV sector. These cells incorporate organic and carbon compounds, such as graphene and carbon nanotubes, which offer a reduced ecological footprint compared to their metal-halide counterparts. Nonetheless, challenges related to the stability, efficiency, and long-term environmental impacts of both M-PSCs and C–PSCs remain.

There is a need for a balanced approach that does not compromise technological advancement for environmental safety. The ongoing research and development efforts are aimed at overcoming these challenges through the development of lead-free and less-toxic perovskite materials, advanced encapsulation techniques to contain hazardous substances and strategies for the recycling and reuse of PSC components.

Recent research in PSCs has led to innovative designs that enhance their application and efficiency across various domains. One notable advancement is the integration of organic small molecules as electron transport layers, which has improved charge transport and overall efficiency, achieving performances up to 18.2 % [5]. This technique optimizes the interface between layers, enhancing charge extraction and minimizing recombination losses. Furthermore, the development of all-inorganic M-PSCs has been pivotal, offering improved thermal stability and durability, making PSCs more robust for commercial use and large-scale deployment. Another significant stride has been made in the area of low-toxicity, lead-free alternatives, with tin-based perovskites being explored to reduce environmental and health impacts, thus potentially increasing market acceptance. Additionally, research has extended into flexible and wearable PSCs, utilizing polymer substrates to create bendable and lightweight solar panels that can be integrated into clothing or portable electronics. Lastly, perovskite-silicon tandem solar cells represent a breakthrough in exceeding the efficiency limits of traditional solar cells by layering perovskite cells atop silicon to harness a broader spectrum of sunlight, significantly boosting overall efficiency. These advancements underscore the dynamic evolution of PSC technology and its growing role in meeting diverse energy needs efficiently and sustainably.

Recent technological and economic progress has triggered an increase in energy consumption worldwide [6]. This heavy dependence on fossil fuels for a myriad of human activities has led to adverse environmental consequences, notably global warming [7]. Predictions suggest that, given the ongoing consumption patterns, fossil fuel reserves might be exhausted by the 22nd century, thereby increasing the urgency to explore renewable energy sources. The Paris Agreement, established during the COP21 conference in Paris on December 12, 2015, under the United Nations Framework Convention on Climate Change (UNFCCC), represents a significant international commitment to address climate change and to promote a shift towards a sustainable, low-carbon future. Its primary objective is to limit global warming to well below 2 °C above pre-industrial levels during this century, with an ambition to further limit the temperature rise to 1.5 °C. To fulfill the objectives of the Paris Agreement, there is a crucial need for the rapid development and deployment of technologies that not only reduce carbon emissions but also actively remove carbon dioxide from the atmosphere, a movement that has drawn significant interest [8].

The field of PV technology has seen remarkable progress recently, fueled by the surge in demand for renewable energy alternatives. The journey began with the pioneering crystalline silicon (c-Si) solar cell introduced by Bell Labs in 1941 [9], leading to continuous advancements through numerous enhancements and the innovation of light-absorbing materials to boost the efficiency of solar cells. PSCs, known for their polycrystalline thin films made from M-PSCs and C–PSCs, have captured significant interest for their role in advancing future solar technologies due to their efficient production costs and superior photoelectric conversion efficiency (PCEs) [[10], [11], [12], [13], [14]].

The evolution of PSCs has marked a significant leap in PCEs, showcasing their promising role in the renewable energy sector. These cells incorporate a perovskite layer for absorbing sunlight, sandwiched between charge transport layers composed of either organic or inorganic substances. This configuration excels at converting sunlight to electricity by facilitating the generation and separation of electron-hole pairs. The versatility in their band gap allows them to harness a broad spectrum of sunlight, while their high carrier mobility and extended diffusion lengths contribute to their elevated PCEs [15]. Offering a cost-effective alternative to traditional silicon-based cells, which rely on more intricate and costly fabrication techniques, PSCs benefit from simpler production methods like spin-coating, inkjet printing, and vapor deposition for applying perovskite materials. This enhances their affordability and paves the way for mass production, further reduced by the readily available and cost-efficient materials used in their construction, such as hybrid organic-inorganic Pb or tin (Sn) halide-based compounds. Notably, the efficiency of PSCs has surged from 3.8 % in 2009 to 29.8 % in 2021, a rapid increase highlighted in Table 1, establishing them as a formidable contender in the solar energy arena [[1], [2], [3], [4]].

**Table 1:** Device efficiency of high-quality perovskite [16].

	Efficiency, Year	Device area	Certification
MicroQuanta Semiconductor	21.4 %, 2021	19.32 cm^2^	JET
UtmoLight	20.1 %, 2021	63.98 cm^2^	JET
Perovs	18.07 %, 2020	46.2 cm^2^	Newport
Panasonic	17.9 %, 2019	804 cm^2^	AIST
Kunshan GCL Optoelectronic Material	15.3 %, 2019	2925.0 cm^2^	TÜV
OxfordPV (tandem)	29.52 %, 2020	1.12 cm^2^	NREL
Helmholtz Center Berlin (tandem)	29.80 %, 2021	1.00 cm^2^	ISE

Swift progress advancements in PSC technology demonstrate its potential to surpass existing solar power methods. The focus is on novel device structures and materials, combining carbon-based components such as graphene, carbon nanotubes (CNTs), fullerenes, and conductive polymers like poly(3,4-ethylenedioxythiophene) polystyrene sulfonate (PEDOT:PSS) and poly(3-hexylthiophene) (P3HT) with M-PSCs films, known for their high light absorption and tunable band gaps. Techniques such as using indium tin oxide (ITO) for semi-transparent cells, employing flexible substrates for wearable tech, and applying uniform deposition methods are key. Innovations also include enhancing light capture and conductivity with silver nanowires and quantum dots, alongside using materials like Spiro-OMeTAD, TiO_2_, and SnO_2_ for effective charge transport. Further improvements with conductive polymers, anti-reflective coatings, and encapsulation methods aim to boost efficiency and longevity. These developments are crucial for elevating PSCs' performance and stability, promoting their adoption of renewable energy.

The merging of materials from C–PSCs and M-PSCs represents a significant advancement in the search for renewable energy technologies, covering uses from effective light harvesting to photodetection. M-PSCs are distinguished into Pb, Sn, and hybrid types, where the perovskite structure substitutes two Pb^2+^ ions with one monovalent and one trivalent ion, enhancing stability and efficiency [17]. Despite the potential of Sn-based PSCs, their susceptibility to environmental degradation remains a concern [18]. In response, the focus has shifted towards safer alternatives like antimony (Sb^3+^) and bismuth (Bi^3+^), aiming to reduce the environmental footprint without compromising too much on performance [19,20]. Conversely, PSCs incorporating carbon elements, such as graphene, present a greener option, addressing the toxicity concerns linked to M-PSCs. While C–PSCs have traditionally lagged in efficiency, ongoing research strives to close this gap, enhancing their viability as a sustainable energy source. This evolving landscape highlights the ongoing challenge of aligning efficiency with environmental responsibility in PSC development.

Currently, PSCs undergo encapsulation to prevent hazardous chemicals from leaking into the environment [21,22]. However, environmental disasters such as storms, landslides, fires, or improper disposal may cause these cells to release toxic substances into nature [[23], [24], [25]]. This release can lead to the contamination of soil and water through rainfall and snowmelt [21]. A notable instance is the complete breakdown of the absorber layer in methylammonium Pb iodide PSCs due to damage from moisture [21]. The escape of Pb or Sn compounds into the surroundings could contaminate soil and water bodies [18,21], be absorbed by plants [26], and pose health risks to humans and animals [18,21,25,26]. The environmental impact of M-PSCs is not limited to metallic pollutants but also includes the release of other harmful toxics (refer to Table 2) during degradation [18]. For example, the level of hydroiodic acid released by CH_3_NH_3_SnI_3_ poses a more significant threat to aquatic life than an equivalent amount of Pb from CH_3_NH_3_PbI_3_ [18]. Similarly, C–PSCs, employing substances like graphene and CNTs, might release these less toxic but still potentially harmful materials into the environment, especially when the encapsulation is compromised by weather events. The deterioration of C–PSCs can lead to the dispersal of carbon nanoparticles into ecosystems, potentially impacting flora and, indirectly, human and animal health.

**Table 2:** Differences in additives for M-PSCs and C–PSCs and their related risks [27].

Additive Name	Chemical Formula	MCL (mg/L)	LD50 (mg/kg)	PEL (mg/m^3^)
Zinc	Zn	NA	NA	NA
Ethanol	CH_3_CH_2_OH	NA	NA	1900
Acetone	CH_3_COCH_3_	NA	5800	2400
bis(2,4-pentanedionato)bis(2-propanolato)titanium(IV)	C_16_H_28_O_6_Ti	NA	NA	NA
1-butanol	CH_3_(CH_2_)_3_OH	NA	790	300
Formamidine acetate	HN=CHNH_2_⋅CH_3_COOH	NA	NA	NA
Hydroiodic acid	HI	NA	NA	NA
Methylamine	CH_3_NH_2_	NA	NA	12
Hydrobromic acid	HBr	NA	NA	10
Ethyl ether	(C_2_H_5_)_2_O	NA	1215	1200
Lead(II) iodide	PbI_2_	0.015	0.5	NA
Lead(II) bromide	PbBr_2_	0.015	NA	NA
N,N-Dimethylformamide (DMF)	(CH_3_)_2_NC(O)H	NA	NA	30
Dimethyl sulfoxide (DMSO)	(CH_3_)_2_SO	NA	>5000	NA
2-isopropanol	(CH_3_)_2_CHOH	NA	5045	980
Isopropanol	CHb_3_CHOHCH_3_	NA	5045	980
Chlorobenzene	C_6_H_5_Cl	0.1	NA	350
Spiro-OMeTAD	C_81_H_68_N_4_O_8_	NA	NA	NA
4-*tert*-butylpyridine	C_9_H_13_N	NA	NA	NA
Li-TFSI solution	Li	NA	NA	NA
Gold	Au	NA	NA	0.01
Graphene	C	NA	NA	NA
CNTs	C	NA	NA	NA
Fullerene	C60	NA	NA	NA
Conductive Polymers (e.g., PEDOT:PSS)	(C_2_H_3_Cl)n	NA	NA	NA
P3HT	(C_6_H_10_S)n	NA	NA	NA

Table 2 showcases an array of organic solvents, metallic elements, and additional chemical compounds, underlining the varied chemical compositions and potential risks tied to the components utilized in constructing M-PSCs. In the realm of C–PSCs, including materials such as graphene, CNTs, PEDOT:PSS, and P3HT, the potential for toxicity significantly depends on factors like the amount, duration of exposure, and the unique properties of the material. The median lethal dose (LD50) values serve as indicators of a substance's immediate toxicity, whereas lower figures suggest a greater level of toxicity. Permissible exposure limits (PELs) set the threshold for the highest concentration of a chemical that employees may be exposed to within a workplace setting. Meanwhile, maximum contaminant levels (MCLs) establish the utmost allowable concentration in drinking water, highlighting issues primarily with Pb-based compounds, though they might not be as pertinent to many additives used in applications beyond consumption.

The progression of PV technology plays a crucial role in the transition to a low-carbon energy framework, yet the high toxicity of certain metals and compounds presents significant environmental and health challenges, hindering the commercial success of PSCs [28,29]. The long-term integrity and durability of perovskite materials also pose major issues, as they are prone to degradation when subjected to humidity, heat, and direct sunlight, leading to a noticeable decrease in performance over time. Particularly, the use of Pb in these materials raises serious safety concerns due to its harmful effects. The risk of extensive soil and water contamination arises from the production, improper disposal, or accidental damage of solar cells containing Pb, a heavy metal known for its severe health and environmental hazards. Moreover, the production of C–PSCs also leaves a considerable environmental footprint that warrants attention. Efforts to enhance their stability and longevity include the development of encapsulation techniques and robust protective layers by researchers aimed at prolonging the operational life of PSCs. Recent studies are exploring safer alternatives like Sn-based or hybrid perovskites to reduce environmental harm while retaining promising optoelectronic properties [[30], [31], [32], [33]].

Yet, there is a clear gap in comprehensive analyses that methodically assess the environmental impact of components used in both M-PSCs and C–PSCs on ecosystems, including soil, plant life, and wildlife, as well as their potential environmental hazards. Moreover, some studies point out the lack of detailed contamination studies in actual environmental conditions, highlighting the urgent need for thorough field research to understand the real effects of PSC implementation and the effectiveness of various mitigation strategies in different ecological contexts. Consequently, this review gathers and scrutinizes recent research on the creation of M-PSCs and C–PSCs and their impact on environmental sustainability, food security, and public health. It also reviews the waste generated from both M-PSCs and C–PSCs, proposing methods and strategies aimed at reducing toxicity by enhancing recycling and reuse efforts.

The manuscript begins by addressing a significant gap in the existing literature, specifically the lack of comprehensive analyses that systematically evaluate the environmental impacts of components used in M-PSCs and C–PSCs on various ecosystems, including soil, plant life, and wildlife. This review is critical as it highlights the absence of detailed contamination studies conducted under actual environmental conditions, underscoring the urgent need for extensive field research to grasp the true effects of PSC deployment. These studies are essential to evaluate the effectiveness of different mitigation strategies tailored to diverse ecological contexts.

## 2 Shining light on solar innovation: M-PSCs and C–PSCs advancements

a. Metal-Halide Perovskite Solar Cells

**Fig. 1:**
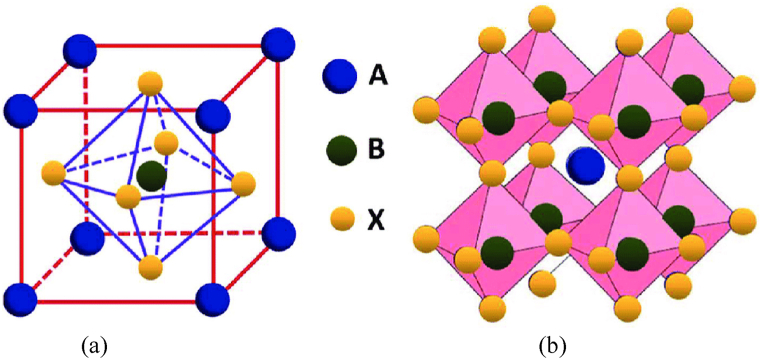
(a) Cubic Perovskite Structure. (b) Polyhedral Representation of Perovskite Structure. The 3D crystalline architecture of perovskite ABX_3_: here, 'A' represents Cs,CH_3_NH_3_, or NH_2_CHNH_2_; 'B' is either Pb or Sn; and 'X' denotes a halide ion [34].

Perovskites are characterized by their ABX_3_ crystal configuration, which typically forms in a cubic structure with an AX_12_ cuboctahedral center linked to a BX_6_ octahedral boundary. Fig. 1 demonstrates a typical perovskite structure depicted through both a simplified cubic lattice and a polyhedral model. These representations emphasize the ABX_3_ configuration prevalent in perovskite materials, where A and B are cations, and X is an anion.

• **Cubic Lattice Representation**

Fig. 1a shows the cubic lattice representation, the red box outlines the unit cell, the fundamental repeating unit in the crystal structure. The blue spheres, positioned at the corners of the cubic cell, represent the A-site cations, which are typically larger than the B-site cations. The green sphere, centrally located within the cube, represents the anion that coordinates with both A and B cations. The yellow spheres, located at the body center of the cube, are the smaller B-site cations that usually have a higher charge density compared to A-site cations. These B cations coordinate closely with the X anions, as indicated by the dashed lines, which perhaps represent bonding interactions and show how the X anions coordinate with both A and B cations.

• **Polyhedral Model**

Fig. 1b provides a polyhedral model where pink polyhedra represent the coordination environment around the B-site cations. Each polyhedron, formed by X anions at the vertices, highlights the octahedral coordination around the B cations. The blue sphere appears at the center of a larger cubic configuration formed by joining the vertices of the surrounding octahedra, indicating a cuboctahedral coordination by the X anions. This model shows a three-dimensional arrangement where multiple BX_6_ octahedra share corners, illustrating how the B cations are linked through the X anion network, leading to a robust structure.

This perovskite structure is noted for its stability and flexibility, which is influenced by the size and charge of A and B cations as well as the X anions. The flexibility allows for various substitutions at A and B sites, which can alter physical properties like ionic conductivity, magnetism, and electronic band structure. The connectivity and the nature of the bonds within perovskites significantly affect their optical, electrical, and magnetic properties, making them suitable for a variety of applications, including solar cells, superconductors, and catalysts.

The stability and morphology of this structure are determined by the Goldschmidt tolerance factor, defined as tolerance factor $t=\tau_{A}+\tau_{X}/\sqrt{2\left(\tau_{B}+\tau_{X}\right)}$, and the octahedral factor, $\mu =\tau_{B}/\tau_{X}$, calculated based on the ionic radii of the ions located at the A, B, and X sites ($\tau_{A}$, $\tau_{B}$, and $\tau_{X}$, respectively). The tolerance factor is a measure used to determine the fit of the A site cation within the BX_3_ framework. A perfect match is indicated by a tolerance factor of exactly 1; a range of 0.8 ≤ t ≤ 1 generally allows for the creation of perovskite structures, though at the lower end, these may show distortions due to the tilting of BX_6_ octahedra and a decrease in symmetry. A value of t > 1 implies that the A site cation is too bulky, typically preventing the formation of a perovskite structure, while a value of t < 0.8 suggests the A cation is too diminutive, frequently leading to the formation of structures other than perovskites. This parameter has been instrumental in identifying and predicting the stability of oxide and fluoride perovskites, specifically ABX_3_ compounds where X equals O^2−^ or F^−^ [35].

The strong electronegativity of these anions contributes to a significant ionic bonding character, supporting the assumptions of the hard-sphere model. In a detailed analysis of ABO3 compounds in 2004, the researchers recorded 192 variations of ABO_3_, out of which 121 were found to form perovskite structures at standard temperature and pressure [36]. Out of these, 163 (or about 85 %) were accurately classified as either perovskites or non-perovskites using the tolerance factor as a criterion for perovskite formation (0.8 ≤ t ≤ 1). Furthermore, their analysis of 65 ABF_3_ compounds showed that 62 (95 %) were correctly identified as perovskites when applying a stability threshold of t > 0.85 [37]. The composition and structure of M-PSCs feature organic monovalent cations like methylammonium (MA) and formamidinium (FA), or alkali cations such as Cs^+^ and Rb^+^, at the A sites. Meanwhile, the B and X sites are occupied by divalent metal ions (e.g., Pb^2+^ and Sn^2+^) and halide ions (e.g., I^−^, Br^−^, and Cl^−^), respectively.

M-PSCs have garnered significant interest since the groundbreaking discovery of hybrid perovskite solar absorbers by Kojima and colleagues in 2009 [38]. These hybrid M-PSCs, with a general formula of ABX_3_, feature a small organic cation, like methylammonium (CH_3_NH_3_^+^), at the A site and a halide anion at the X site. Notably, these materials demonstrate exceptional light absorption and charge carrier separation capabilities, yet they can be synthesized using straightforward, low-tech chemical processes, positioning them at the forefront of novel solar cell technologies [[39], [40], [41]]. Iodide-based perovskites, particularly CH_3_NH_3_PbI_3_ with a band gap of approximately 1.5 eV, stand out for their efficiency, closely matching the ideal band gap for single-junction PV cells [42].

Since their introduction in 2009, PSCs have made remarkable strides, with their PCE increasing from an initial 3.8 % to over 25 % in just a decade. This impressive progress results from meticulous material design, precise control over perovskite crystallization, and effective carrier suppression. Another significant advantage of PSCs is their adaptability for use in flexible solar cells, which is facilitated by the ability to produce high-quality thin films through low-temperature solution-based processes. This adaptability also allows for the adjustment of the bandgap, enabling seamless integration into tandem configurations with commercially available silicon solar cells, leading to varied and impressive PCE outcomes in configurations such as single-junction, flexible, and perovskite-silicon tandem solar cells.

**Fig. 2:**
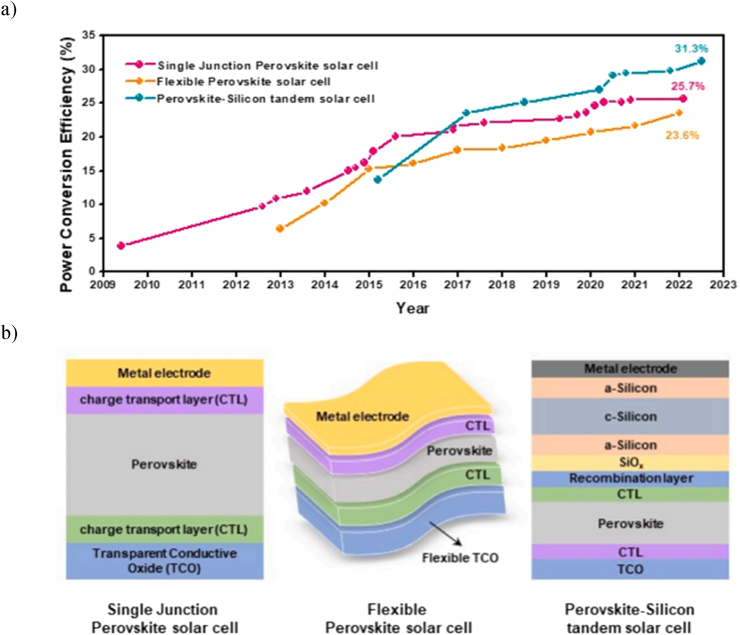
(a) Trends in PCE across different PSC variants. (b) configurations of single-junction, flexible, and perovskite-silicon tandem PSCs [32,36,38,43].[32,36,38,43]. (For interpretation of the references to colour in this figure legend, the reader is referred to the Web version of this article.)

Fig. 2a illustrates these efficiency trends through a line graph, which tracks the PCE of three distinct types of PSC from 2009 to 2023. The single-junction PSC displays a steady rise in efficiency, peaking at approximately 31.3 % by 2023, represented by a pink dotted line. The Flexible PSC also shows a significant increase in efficiency over time, marked by a teal line, and reaches about 25.7 % by 2023. The perovskite-silicon tandem solar cell, indicated by an orange line, exhibits the most dramatic efficiency growth. Starting from around 2014, it surged to about 23.6 % by 2023. This graph effectively highlights the advancements and performance trends among the different types of PSC over the studied period.

Fig. 2b presents a comparative analysis of the structural design of three different types of PSCs:

• The *single junction PSC* consists of several layers, starting with a transparent conductive oxide (TCO) at the base, acting as a transparent electrode. Positioned above the TCO is the charge transport layer (CTL), which aids the movement of electric charges within the cell. The core of the cell is the perovskite layer, where sunlight is absorbed and converted into electrical charge, topped with a metal electrode that collects and transfers the electrical charge.
• The *flexible PSC*, constructed similarly to the single-junction model, includes a flexible substrate that allows the cell to bend. This flexibility is achieved by using a flexible TCO and adjusting the layering to maintain performance under bending conditions.
• The *perovskite-silicon tandem solar cell* enhances efficiency with additional layers. It begins with a TCO and a CTL, similar to other models. The perovskite layer in this cell is specially optimized to complement an additional silicon layer. Notably, this model includes a recombination layer and an SiO_2_ layer, crafted to manage charge recombination and separation between the perovskite and silicon layers effectively. Below these layers, the a-Silicon and c-Silicon layers absorb various light spectra, significantly enhancing the cell's overall efficiency. The structure concludes with a metal electrode, consistent with the other models, to facilitate charge collection and transfer.

Each design showcases the technological advancements in PSC technology, tailoring structural components to meet specific performance criteria and application demands, thus demonstrating the potential and versatility of PSC in contemporary solar technology applications. These achievements underscore the versatility of PSCs, extending their applicability beyond traditional PV systems to include innovative uses in building-integrated photovoltaics (BIPV) and vehicle-integrated photovoltaics (VIPV) systems. To date, first-generation silicon-based solar cells have dominated the market due to their impressive PCE and robust longevity. Alterations in the halide component or the exploration of different ABX_3_ structures, like hexagonal perovskite, tend to increase the band gap, which inversely affects PV efficiency. Before the advent of hybrid solar cells, both hybrid and purely inorganic iodide perovskites were the subject of extensive research due to their potential in various applications [[44], [45], [46], [47], [48], [49]].

The remarkable advances in PSC technology have been predominantly anchored in Pb-based formulas, attributed to their exceptional optical and electrical features, such as reduced trap site densities, prolonged carrier diffusion lengths, diminished exciton-binding energies, increased absorption capabilities, and notable dielectric constants [[50], [51], [52]]. Nonetheless, the utilization of Pb, a recognized toxic heavy metal capable of migrating across soil and aquatic environments and accumulating in living tissues, presents significant barriers to the widespread deployment of Pb-based solar solutions [53]. The environmental and health concerns linked to Pb's propensity for bioaccumulation and mobility have considerably restricted the acceptance of Pb-based solar innovations. Known for its detrimental effects on both ecological systems and human health, Pb contamination poses pronounced risks, especially in scenarios where it could directly impact human and environmental well-being. The hazards associated with Pb, including potential neurological and systemic health issues, emphasize the imperative demand for the development of safer solar technologies. The limitations posed using Pb not only constrict the growth prospects of Pb-based PV solutions but also accentuate the essential need for research into alternative materials that offer a balance between safety and operational efficacy.

Consequently, there's been a pivot towards exploring alternatives with metal cations that are less harmful than Pb despite these alternatives typically offering reduced efficiencies [54]. Sn emerges as a leading candidate to replace Pb in PSCs, sharing similar ionic radii and belonging to the same group of 14 elements. Sn-based formulas are advantageous due to their optimal optical bandgap, estimated to be between 1.1 and 1.4 eV, aligning with the maximum theoretical efficiency predicted by the Shockley–Queisser limit (33.7 % at 1.34 eV). However, Sn's lower oxidation state stability compared to Pb poses challenges, with Sn^2+^ easily oxidizing to Sn^4+^ in the presence of air, thus necessitating nitrogen-protected processing environments for Sn-based PSCs, which compromises their performance in open air [55]. Moreover, concerns regarding Sn's toxicity highlight the necessity for environmentally safe and health-conscious technologies for PSC deployment.

The absorption layers in reported PSCs typically measure around 550 nm in thickness. For Pb-based perovskite films, the Pb concentration per unit area is roughly 0.75 g/m^2^, significantly exceeding that found in conventional Pb-based paints (0.007 g/m^2^) [21,56]. Nonetheless, the toxic nature of heavy metals like Pb and Sn mandates the development of effective solutions to mitigate their release into the environment.

b. Carbon-Based Electrode Perovskite Solar Cells

C–PSCs represent a significant shift from traditional M-PSCs towards more environmentally benign materials for PV applications. Unlike their M-PSCs counterparts, C–PSCs utilize organic and carbon materials such as graphene, CNTs, fullerene, conductive polymers like PEDOT:PSS, and P3HT, which serve various roles within the solar cell structure, including as the light-absorbing layer, electron or hole transport layers, and electrodes. These C–PSCs do not follow the ABX_3_ crystal structure typical of perovskite materials. Instead, their structural and electrical properties are derived from the arrangement of carbon atoms and the incorporation of organic molecules. The electronic properties of these materials can be tuned through molecular engineering, allowing for the optimization of the solar cell's performance. For example, graphene, known for its exceptional conductivity and mechanical strength, can be used as an electrode material. Similarly, CNTs and fullerene derivatives are employed for their ability to transport electrons efficiently, while conductive polymers like PEDOT:PSS and P3HT are widely used for hole transport.

Incorporating carbon black/graphite as an anode in PSCs initially led to a PCE of 3.0 % [57,58]. Subsequently, low-temperature cured carbon electrodes, serving as noble metal substitutes in HTL-free PSCs, achieved a PCE of 13.8 %, further improved through optimization via the doctor-blading technique. An innovative method for preparing these electrodes under high humidity resulted in low sheet resistance and excellent substrate adhesion [59]. Additionally, a room-temperature solvent-exchange process produced self-adhesive, macroporous carbon electrodes, reaching a record PCE of 21.0 % for C–PSCs.

**Fig. 3:**
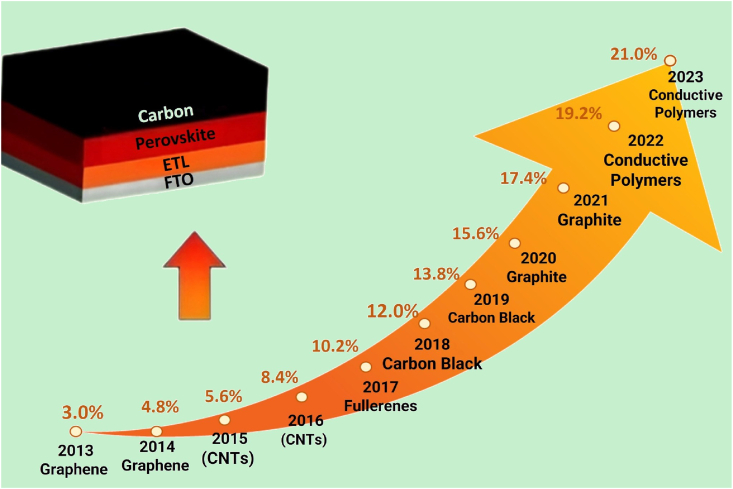
Progression of PCE in C–PSCs: 2013–2023.

Fig. 3 provides a detailed graphical representation of the evolution and improvement in PCE of PSC from 2013 to 2023, with a particular focus on the advancements in the materials used for the electron transport layer (ETL) [37,43,[60], [61], [62]]. As shown in the figure, doping C–PSCs has significantly enhanced efficiency. This improvement is attributed to the dopants altering the electrodes' conductivity and work function, optimizing the cells' overall performance. The structure of a typical PSC is depicted at the base of the visual, illustrating the layer composition from bottom to top:

• *FTO (Fluorine-doped Tin Oxide)*: This bottom layer acts as the transparent conductive oxide and serves as the base electrode.
• *ETL*: Positioned above the FTO, this layer is crucial for the movement of electrons and plays a vital role in enhancing the solar cell's efficiency.
• *Perovskite Layer*: This is the active layer where sunlight is absorbed and converted into electrical energy.
• *Carbon*: This top layer is likely a representation of the carbon-based counter electrode or part of the encapsulation or interface layers, though not specified in detail.

The progression of materials used in the ETL is highlighted by an arrow and associated timeline, indicating significant transitions over the years:

• *2013–2015*: The use of Graphene and Carbon Nanotubes (CNTs) marked the initial materials for the ETL, starting with efficiencies at 3.0 % in 2013 and rising to 5.6 % by 2015.
• *2016–2019*: A shift to fullerene and carbon black was noted, with a substantial improvement in efficiency from 8.4 % in 2016 to 13.8 % in 2019.
• *2020–2022*: Graphite and conductive polymers were introduced, pushing the efficiency further to reach up to 19.2 % by 2022.
• *2023*: The efficiency peaked at 21.0 % with the advanced use of conductive polymers, marking the highest efficiency achieved within the decade.

The infographic effectively underscores the significant advancements in material technology for the ETL in PSCs. These innovations have directly contributed to the enhanced efficiency of these cells over a decade. This progression not only demonstrates the rapid development within perovskite solar cell technology but also highlights the potential of continuous material innovation in extending the boundaries of solar energy efficiency.

The absence of heavy metals such as Pb or Sn in C–PSCs significantly reduces the potential toxicity and environmental hazards associated with the solar cell's lifecycle, from production to disposal. However, the stability and durability of C–PSCs under environmental conditions such as moisture, heat, and light exposure remain areas of ongoing research. Innovations in material science and engineering are continually improving the stability and efficiency of C–PSCs, to achieve performance comparable to or exceeding that of M-PSCs. While C–PSCs currently do not reach the same efficiency levels as M-PSCs, their development has been driven by the promise of lower toxicity and environmental impact. For instance, the efficiency of C–PSCs has seen significant improvements, with researchers exploring various configurations and composite materials to enhance light absorption, charge transport, and overall cell performance.

## 3 Environmental impacts of M-PSCs and C–PSCs

### 3.1 Contamination of soil

a. M-PSCs on Soil Contamination

Soil contamination concerns with M-PSCs arise from the leakage of hazardous materials like Pb from the perovskite layer. Degradation or improper disposal increases the risk of these substances entering the soil, posing environmental and health hazards. Studies show that encapsulated Sn perovskites are less stable than Pb counterparts, with both types subject to degradation under atmospheric conditions. The breakdown products, including PbI_2_, PbBr_2_, and Sn compounds, can lead to soil acidification and increased metal mobility. Research indicates the need for prompt action following PSC damage to mitigate contamination risks, underscoring the importance of understanding long-term soil health impacts and developing strategies to reduce contamination.

**Fig. 4:**
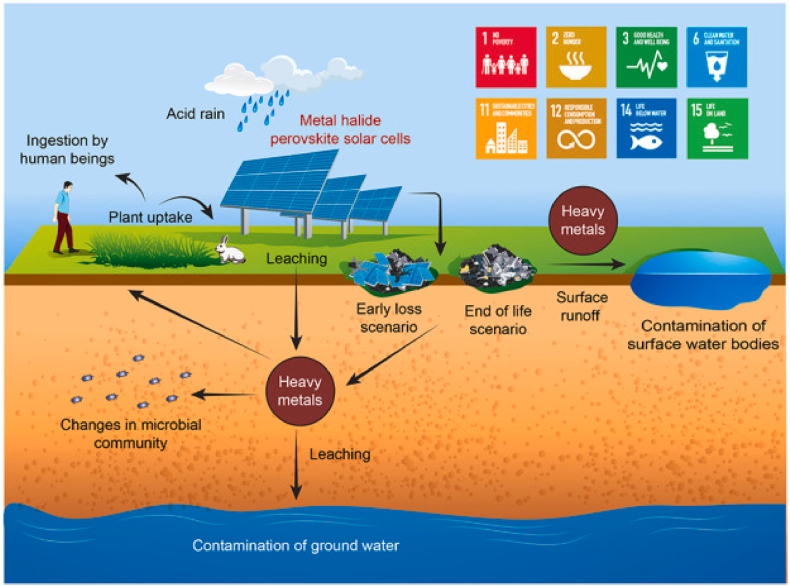
Environmental transport and Fate of heavy metals from PSCs [67].

Perovskites containing Sn are notably less robust than those with Pb, even when encapsulated, as highlighted by Ref. [63]. This issue is compounded by the inherent instability of Pb-based PSCs, which are prone to rapid degradation in atmospheric conditions, as noted by Ref. [64]. The instability of Sn_2_^+^ in the presence of air, due to its oxidation to Sn_4_^+^, causes swift degradation of the film and failure of the device, often within a very short time frame [65]. Consequently, a significant number of these solar cells are discarded at various stages of their lifecycle, either during production in soon after deployment, often due to damage from environmental events or accidents [64]. Despite encapsulation efforts to prevent environmental contamination, breaches in the protective layers can lead to the leakage of hazardous substances into the surroundings through precipitation [25]. Such leakages can introduce toxic elements into the soil, posing a threat to the ecosystem and raising concerns about bioaccumulation risks to humans and wildlife as illustrated in Fig. 4 [66].

Fig. 4 offers a detailed visualization of the environmental impact associated with metal halide PSCs, emphasizing the significant aspects of their use and the subsequent environmental risks, particularly the release and migration of heavy metals. The diagram highlights the critical need for sustainable lifecycle management practices in the development of solar technologies to mitigate these environmental impacts and support global sustainability efforts.

#### 3.1.1 Source of contamination

The central element of the diagram is the metal halide PSCs. These cells, when exposed to environmental factors such as acid rain, can degrade and release heavy metals into the environment.

#### 3.1.2 Environmental pathways

• Leaching: Heavy metals leach into the soil, potentially reaching the groundwater. This can occur directly under the solar cells or via nearby waste disposal sites.
• Plant Uptake: Metals absorbed into the soil can be taken up by plants, which are then ingested by humans and animals, introducing these contaminants into the food chain.
• Surface Runoff: Heavy metals can also enter surface water bodies through runoff, leading to the contamination of lakes, rivers, and streams.

#### 3.1.3 Impact scenarios

• Early Loss Scenario: This refers to the premature failure of solar cells, which can lead to an accelerated release of heavy metals.
• End of Life Scenario: Even under normal operation, the disposal of solar cells at the end of their life can result in heavy metal contamination if not properly managed.

#### 3.1.4 Consequences

• Contamination of Ground and Surface Waters: Both groundwater and surface waters can become contaminated, posing risks to aquatic life and water quality.
• Changes in Microbial Community: The presence of heavy metals can alter soil microbial communities, potentially disrupting soil health and function.

Upon degradation, perovskites revert to their base materials. For Pb-based PSCs, this includes the formation of PbI_2_ or PbBr_2_, traces of metallic Pb, and carbon-based compounds, which can further degrade into hydroiodic acid (HI) and methylamine. SnI_2_ and SnI_4_ are identified as breakdown products of Sn-based cells. The released Pb tends to bind strongly to soil, usually remaining within the upper soil layers. HI, a byproduct, acidifies the soil, enhancing the leaching and movement of metal ions and halides, thereby increasing contamination levels [18]. The bioavailability of Pb from these PSCs is notably higher than from other sources of Pb pollution, suggesting a heightened risk to biological entities, although this risk varies with soil composition [68]. The Pb mobility in the soil is influenced by factors such as pH, clay content, and the presence of water, iron, and manganese oxides, as well as organic matter, with higher mobility observed in acidic conditions. Pb also interacts with secondary soil minerals, forming compounds like anglesite, pyromorphite, and cerussite, which may limit its dispersal [69]. Furthermore, soil redox conditions play a role in Pb mobility, with the reduction of sulfates to sulfides under anaerobic conditions leading to the formation of PbS, thus reducing Pb bioavailability [70].

In a study [64], the release of metals from crushed thin-film solar panels (TFSPs) mixed with various soils showed an increase in concentrations of metals such as Ga, Zn, Pb, Cu, Ni, In, and Cr, with higher TFSP concentrations exacerbating metal release, especially in acidic soils. The presence of heavy metals in soil can disrupt the microbial ecosystem, affecting more adversely fungal populations than bacterial ones [71,72]. At sites with electronic waste, the co-presence of organic pollutants and heavy metals leads to competitive adsorption and reduced degradation of organic compounds due to inhibited microbial metabolism [73]. The impact of heavy metals on soil microbes also depends on the soil's physical and chemical characteristics.

Recent findings suggest that even after significant damage to well-encapsulated PSCs, swift action can mitigate Pb contamination in soil, with one study noting a 3.4 ppm increase in Pb levels below a damaged solar module, advocating for prompt intervention post-incident [74]. This indicates that the potential environmental risk posed by M-PSCs may be lower than previously assumed. Nonetheless, further research is imperative to understand the long-term consequences of various compounds in M-PSCs on soil health and to develop effective contamination reduction strategies.

b. C–PSCs on Soil Contamination

The concern regarding the toxicity of carbon-based materials, such as graphene and CNTs, to soil organisms is significant. Unlike the acute toxicity associated with heavy metals found in Metal-PSCs, these materials do not show immediate toxic effects. However, their prolonged presence in soil environments raises questions about their long-term impact on soil health. The interaction of carbonaceous materials with soil components is complex and influenced by several factors, including the size, shape, and functionalization of the nanoparticles, as well as the physicochemical properties of the soil. The mobility of these materials in the soil determines their potential to cause contamination. For instance, smaller nanoparticles may be more mobile and, hence, more likely to be taken up by plants or leach into groundwater, posing risks beyond the immediate site of contamination [[75], [76], [77], [78]].

Graphene and its derivatives undergo partial degradation, leading to the release of smaller fragments that maintain the chemical inertness and high surface area of the original material. These fragments have the potential to absorb soil nutrients and contaminants, potentially immobilizing them. Similarly, CNTs, crucial to C–PSCs, present a risk with their needle-like structure capable of penetrating soil aggregates and altering soil's physical makeup. CNTs also interact with soil microorganisms, potentially suppressing beneficial microbial species or changing the microbial community structure due to their antimicrobial traits. Fullerene derivatives, resulting from the degradation of solar cells, tend to accumulate in the soil, where their hydrophobic nature leads them to bind with organic matter, possibly impacting soil water retention and nutrient distribution. Conductive polymers break down into various smaller organic compounds that, depending on their makeup, might be bioavailable and potentially toxic to soil organisms, thus affecting overall soil health and functionality.

Recent studies have explored how CNTs characteristics, including size, surface treatment, exposure amount, and duration, affect their toxicity [78]. Evidence points to their negative environmental impacts and disruptive effects on soil microbial ecosystems, potentially decreasing enzyme activities and microbial biomass [[79], [80], [81], [82], [83], [84], [85]]. Short-term exposure to multi-walled carbon nanotubes (MWCNTs) has been shown to significantly impact soil enzyme activity and microbial biomass, especially at higher concentrations [86]. Similar outcomes were observed with single-walled carbon nanotubes (SWCNTs) [84], with a study indicating that SWCNTs might alter microbial community structures in activated sludge [87]. Functionalized MWCNTs (fMWCNTs) are found to exert a more significant impact on microbial life due to their improved dispersibility and direct microbial interactions, suggesting intensified effects at higher concentrations.

Graphene nanomaterials (GNMs) have been found to significantly affect the concentrations of various ions in the soil, with their impact observed in the order of sulfate, phosphate, ammonia, and nitrate [75]. Such variations are critical as they directly affect soil fertility, enzyme activities, and the health of microbial communities, which are vital for nutrient cycling within the soil ecosystem. Alterations in the concentrations of sulfate and phosphate, for instance, can upset the soil's nutrient balance, potentially impacting plant growth and microbial functions. The study in Ref. [75], explored the effects of GNMs on soil by conducting column experiments with both agricultural and undisturbed soils. These experiments utilized concentrations of 10 mg/L and 200 mg/L of GNMs to assess their impact.

**Fig. 5:**
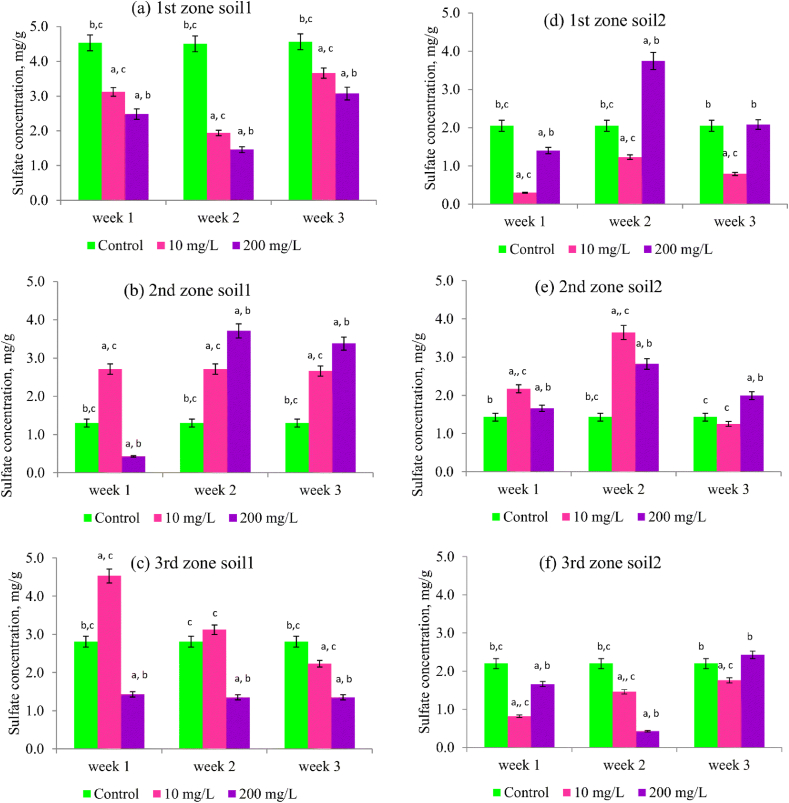
Variation in sulfate levels in soil columns exposed to GNMs concentrations of 10 and 200 mg/L. Statistical significance denoted by differing letters: (a) compared to control (p < 0.05), (b) compared to 10 mg/L GNMs (p < 0.05), (c) compared to 200 mg/L G NMs (p < 0.05) [75].

Fig. 5 visually represents the influence of these GNMs on soil sulfate levels using a series of bar graphs. These graphs show sulfate concentrations measured across three different soil zones over a three-week period. Each graph, labeled from (a) to (f), corresponds to a specific soil sample subjected to varying treatment conditions: a control group without treatment and groups treated with 10 mg/L and 200 mg/L of GNMs. Annotations on the bars with different letters signify statistically significant changes in sulfate levels, highlighting the distinct impact of GNMs on soil health. This visualization not only underscores their effects on soil pH and nutrient availability but also the broader environmental implications for soil ecosystems.

• *Variability Across Zones and Treatments*: The sulfate concentrations vary across the three zones and two different soil samples within each zone, indicating that soil characteristics or environmental conditions might influence sulfate levels.
• *Treatment Impact Over Time*: In most panels, the sulfate concentration changes over the three weeks, generally showing different trends for each treatment level. This suggests that the substance applied might have a time-dependent effect on sulfate accumulation in the soil.
• *Comparison Between Concentrations and Controls*: In many cases, the treated samples (both 10 mg/L and 200 mg/L) show higher sulfate concentrations compared to the control, particularly by the third week. This increase might indicate that the substance used in the treatment could be contributing to greater sulfate accumulation.
• *Statistical Significance*: The graphs are annotated with letters (a, b, c, etc.), which likely denote statistical significance between different treatment levels at each time point. Consistent lettering across a single bar suggests no significant difference at that concentration across weeks, whereas different letters indicate statistically significant changes.

The application of the tested substance has an observable impact on sulfate accumulation in soil, with variations depending on the zone, soil type, concentration of the substance, and duration of exposure. The results highlight the importance of considering both dosage and time when assessing the environmental impact of soil treatments on sulfate dynamics.

### 3.2 Contaminants on surface and groundwater ecosystems

Solar cell contaminants can permeate the soil, reaching groundwater via leaching and surface water through runoff, potentially surpassing safe limits and causing metal toxicity in water-dwelling and land organisms, as indicated by Ref. [88]. The environmental impact, particularly the leaching potential of PSCs, remains underexplored. Sn-based PSCs, for instance, have shown a greater effect on freshwater ecotoxicity compared to Pb-based PSCs, demonstrating that Pb-based PSCs, when discarded in landfills, leach 70 % of Pb within the first year, although 98 % of Pb can be recovered through incineration [89]. PSCs are considered hazardous when Pb concentrations surpass the USEPA's 5 mg/L limit for hazardous waste, and they pose significant risks to water quality by increasing organic carbon and chemical oxygen demand in their leachates. Additionally, initial leaching cycles of PSC powder show a notable decrease in both total organic carbon and chemical oxygen demand, from 2.41 to 60.2 mg/L to 0.13 and 15.08 mg/L, respectively, indicating a degradation in water quality due to pollutants from these solar cells [27]. Notably, a study in Ref. [21] conducted experiments showing substantial Pb losses in perovskite materials under conditions mimicking acid rain. The study found that the solubility of methylammonium-lead-iodide-based perovskites was significantly affected by the pH of simulated rain, with Pb losses reaching 71.9 % ± 4.1 %, 67.5 % ± 5.7 %, and 69.0 % ± 4.5 % at pH levels of 4.2, 6.0, and 8.1, respectively. This indicates a pronounced impact of acid rain's composition on the rate at which Pb is leached from these materials.

Environmental issues also affect copper indium gallium diselenide (CIGS) solar technologies, which have the potential to release cadmium (Cd) and molybdenum (Mo) beyond the limits recommended by the World Health Organization (WHO), depending on environmental conditions. Specifically, for both humid and arid environments, the released levels of Mo and Cd were significantly higher than the WHO's safety limits of 70 μg Mo/L and 3 μg Cd/L [90]. The implications observed in CIGS cells regarding environmental risks may also hold for PSCs. This situation highlights the potential risk of nanotoxicity due to the emission of nanoparticles from these cells, underscoring the need for further research and regulation to mitigate these environmental risks. PbI_2_ and SnI_2_ impacts on zebrafish, as studied in Ref. [91], highlight potential acute toxicity, with SnI_2_ showing greater lethality. Moreover, perovskite nanomaterials' effects on aquatic life, like Daphnia magna, reveal varying toxicity levels, underscoring the necessity for further investigation into PSCs' environmental impacts, especially concerning water contamination [92,93].

## 4 PSCs potential impact on human health

Assessing human health risks associated with PSCs is complex, mainly because it often relies on the 'read-across method,' which extrapolates data from similar substances in various contexts like soil particulates and industrial byproducts. This method reveals a significant gap in specific data about the bioavailability and impacts of toxins directly from PSCs. Human exposure to toxins from PSCs, particularly when substances like methylamine or metal halides (e.g., PbI_2_, PbBr_2_) are released into the environment, is uniquely harmful and potentially more severe than exposure to common soil and dust [94]. The health risks from these substances are largely determined by their solubility and how easily they can be absorbed by the body, which environmental conditions like pH and redox levels can affect.

The use of organic solvents in PSC manufacturing poses notable health risks. These solvents dissolve and blend with precursor materials but are known for their high toxicity, particularly in the production of high-efficiency PSCs [95,96]. Solvents, divided into categories like precursor solvents, antisolvents, and those used in the ETL and HTL, present significant toxicity risks that hinder PSC technology's broader acceptance and use. The choice of solvents directly impacts the efficiency of ETL and HTL, affecting the overall performance of PSCs [97]. Some solvents, including isopropanol, are recognized for their potential to irritate the respiratory system and skin [98]. The European Chemical Agency (ECHA) has labeled these solvents as substances of very high concern (SVHC) due to their toxicity [99]. Although life cycle analysis (LCA) is a standard assessment tool for gauging the environmental and health impacts of technologies, specific concerns related to the solvent used in PSC fabrication, such as the enhanced bioavailability of Pb, leading to increased risks of skin and oral exposure, have not been fully explored [100]. This oversight underscores the need for a more detailed investigation into the health implications of solvent use in PSC production.

The toxicity of Pb and Sn in PSCs is a significant concern due to their links to serious health problems, such as cognitive issues in children and organ failure in adults [101]. These effects are exacerbated by the metals' forms, how they dissolve, and changes in the environment. It is estimated that generating 2400 GW of electricity with PSCs would require approximately 1.1 % (17,000 tons) of the yearly consumption of Pb in the U.S. market, totaling 1.6 million tons [102]. The environmental release of Pb, even in small quantities, poses grave risks due to its acute and chronic health impacts, particularly on children [103]. Low-level Pb exposure can cause significant accumulation in biological systems, leading to toxicity in both flora and fauna, including humans [104,105]. Research indicates that blood Pb levels below 5 μg/L can negatively impact children's cognitive and physical development, highlighting the dangers of early-life Pb exposure [106].

The use of Pb in the manufacturing of PSCs raises concerns due to its toxic characteristics and the potential risks it poses to the environment and health. Contamination from Pb can cause irreversible damage to soil and water ecosystems, impacting the well-being of humans, animals, and plants [107]. The perovskite layers within PSCs, which are prone to breaking down, may dissolve in water, leading to the release of toxic Pb^2+^ ions into the environment and possibly entering the food chain [108].

**Fig. 6:**
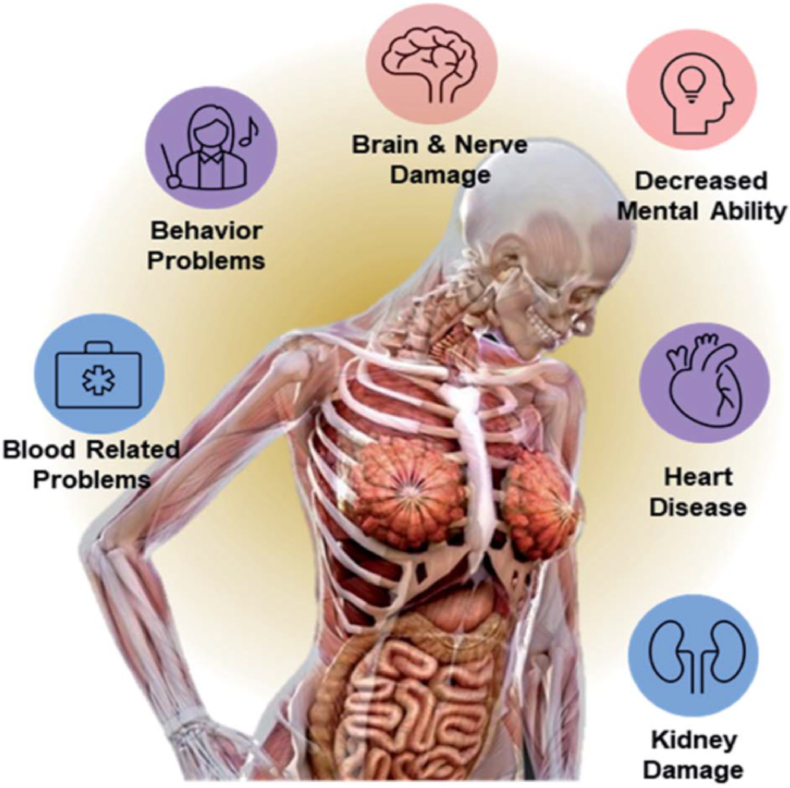
Harmful effects of Pb on the human body [107].

Fig. 6 provides a detailed visual representation of the detrimental effects of Pb exposure on the human body, highlighting a spectrum of health issues affecting various organ systems. At the center of the image is a semi-transparent human figure, strategically annotated to display the major organs, making it easy to understand where and how lead impacts the body's physiology.

This central figure is encircled by several icons, each representing different health concerns associated with lead toxicity:

• *Brain and Nerve Damage*: Lead exposure is shown to cause significant neurological impacts, which include damage to brain and nerve functions.
• *Decreased Mental Ability*: The figure indicates that lead can impair cognitive functions, leading to decreased mental abilities.
• *Behavior Problems*: There is an association between lead exposure and behavioral issues, which could include changes in social behavior and increased aggression.
• *Blood-Related Problems*: Lead also affects the blood's ability to carry oxygen, leading to various hematological issues.
• *Heart Disease*: Cardiovascular problems, including an increased risk of heart disease, are linked to lead exposure.
• *Kidney Damage*: The kidneys can be damaged by lead, affecting their ability to effectively filter and clean the blood.

Each icon not only signifies a specific health risk but also serves to inform viewers of the interconnectedness of these issues, emphasizing that lead's impact is both broad and profoundly detrimental to overall health. The layout and design of Fig. 6 effectively communicates the critical message about the widespread and severe implications of lead exposure in an accessible and visually engaging manner.

Exposure to Pb typically comes from consuming contaminated drinking water or food, emphasizing the importance of establishing regulatory standards to limit Pb levels in environmental resources. Removing Pb from the body proves challenging, as it tends to accumulate in the bones, accounting for about 90 % of the body's total Pb content and has a long half-life of 20–30 years. Pb can disrupt enzyme and receptor functions, inhibit the synthesis of hemoglobin, and lead to the formation of insoluble lead phosphate (Pb_3_(PO_4_)_2_) in the bones. It may also be transferred through breast milk, introducing further risks to infants. The symptoms of Pb exposure can include neurological issues, digestive disturbances, and reproductive problems [109]. The creation of ABX3-based PSCs inherently involves the use of various toxic substances, including dangerous organic ions and halogens, mainly due to the reliance on Pb-based perovskites [110,111]. This highlights the necessity for prudent management and consideration of the materials used in PSC technology to reduce health and environmental risks.

The necessity of incorporating halogens at the X-site within PSCs contrasts sharply with the selection of cations. While indispensable, these halogens, particularly when forming organic halides, introduce significant health hazards. Known for their environmental impact, these compounds necessitate careful management in the rollout of PSC technology. The designation adsorbable organic halogen (AOX) serves to quickly identify the total organic halogens capable of adsorption onto activated carbon, highlighting the carcinogenic, cytotoxic, and genotoxic nature of these compounds [112]. AOX's ability to bioaccumulate and disperse across aquatic systems underscores the indirect risks it poses to human health, prompting the adoption of strict regulatory measures globally. The application of pseudohalide additives like thiocyanate in PSCs aims to improve performance and manufacturing processes. Nevertheless, the environmental persistence and health implications of thiocyanate, despite being less harmful than cyanide, call for a prudent approach to AOX application within PSCs, given its environmental and health repercussions.

The health impacts of Sn are less explored than those of Pb despite Sn's environmental prevalence. No safe exposure level for Sn has been established, with its inorganic forms known to inhibit the absorption of essential minerals like iron, zinc, and calcium due to inefficient absorption and fast excretion [113]. Acute exposure to Sn can cause gastrointestinal distress, such as nausea [114], and long-term exposure is associated with an increased risk of diabetes and obesity [115]. Sn is mainly eliminated through urine, and its toxicity varies by age and compound form, with organic Sn compounds potentially disrupting endocrine and renal functions [113,116].

Sn is considered a safer alternative to Pb in certain perovskite formulations, though its safety is only relative, as it still presents health risks. The toxicity level varies depending on the Sn compound; while inorganic Sn is rapidly expelled from the body, large doses can be detrimental. Organotin compounds employed in Sn-based perovskites are associated with neurological, gastrointestinal, and respiratory problems [117]. The decomposition of Sn-based perovskites results in the formation of SnO_2_ and SnI_4_, with the latter reacting with water to generate respiratory irritants and toxins harmful to aquatic life. Cesium (Cs), utilized in PSCs, may lead to skin burns and respiratory discomfort because of its corrosive nature. The National Institute for Occupational Safety and Health (NIOSH) advises keeping exposure to cesium hydroxide under 2 mg/m^3^ to prevent these hazards, emphasizing the importance of meticulous handling, particularly due to its explosive reaction with water [118].

Some PSCs formulations incorporate chromium (Cr), with its toxicity varying significantly based on its oxidation state. Cr^3+^ has limited absorption by the human body, contrasting with Cr^4+^, which is more readily absorbed and can lead to respiratory problems, including asthma and lung cancer, and oral exposure may cause severe liver and gastrointestinal damage [119]. The breakdown of PSCs releasing methylamine represents another health hazard, impairing kidney function and potentially causing central nervous system issues due to its classification as a uremic toxin. The solubility and environmental reactivity of PSC components, influenced by pH and redox conditions, are critical in evaluating their health risks, highlighting the importance of environmental lifecycle analysis in risk assessments.

The introduction of innovative materials like formamidinium in advanced PSCs brings new concerns over their toxicological effects. Despite extensive research on the toxicity of primary elements like Pb and Sn, the health impacts of newer additives are less known, emphasizing the need for ongoing toxicological studies [120]. Additionally, while halogens used in PSCs, such as iodine and bromide, are generally considered less hazardous, they can form toxic compounds under specific conditions, like bromide producing highly toxic substances in certain environments [121]. This scenario underlines the essential need for thorough research and data gathering on the environmental and health effects of both established and emerging materials in PSC technology to ensure its safe deployment.

## 5 Potential risk mitigation and disposal strategies

### 5.1 Sequestration

Numerous studies have introduced the idea of incorporating a polymer layer within solar modules that captures and holds Pb, preventing its release into the environment. For instance, the Pb leakage was significantly lowered by 62 times through the addition of a cation-exchange resin on the module's glass layer. In 2020, a significant reduction in Pb leakage, by a factor of 25, was achieved through the application of a phosphonic acid-based film on the front glass coupled with a polymer film containing lead-binding agents on the metal electrode side [122]. These films were effective even after exposure to synthetic rain at acidic pH, at both ambient and elevated temperatures. This strategy of Pb capture and containment is seen as a valuable measure to prevent the release of Pb from the modules.

Additionally, the resilience of a PSC module was enhanced against physical damage by encasing it in an epoxy resin capable of self-repair at around 42 °C. This innovation was tested by dropping metal balls on the modules to mimic hail damage, demonstrating that the epoxy layer effectively minimizes Pb release during leaching tests. The self-healing property of the epoxy is particularly beneficial for repairing damage from harsh weather, thus potentially reducing risks to individuals handling the modules post-damage [123].

**Fig. 7:**
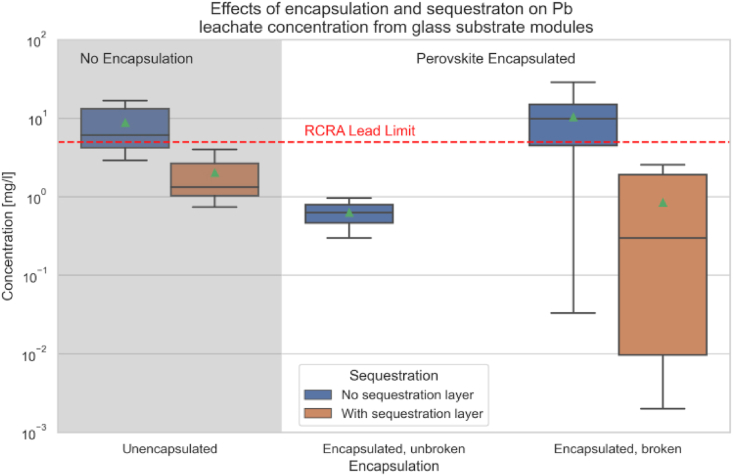
Leaching data of glass substrate perovskite cells and modules with and without encapsulation [124].

Fig. 7 presents a comprehensive analysis of Pb leachate concentrations from glass substrate perovskite solar cells (PSCs) under various conditions, highlighting the impact of encapsulation and the inclusion of a sequestration layer. The concentration of lead is quantified in milligrams per liter (mg/L) and displayed on a logarithmic scale on the y-axis, which helps to delineate variations across a wide range of values.

#### 5.1.1 Comparison groups

• Unencapsulated: Shows lead concentrations from perovskite modules without any protective encapsulation.
• Encapsulated, Unbroken: Displays lead concentrations from intact encapsulated modules.
• Encapsulated, Broken: Represents lead concentrations from broken encapsulated modules.

#### 5.1.2 Sequestration layer

• The graph differentiates samples with no sequestration layer and those with a sequestration layer designed to trap or immobilize lead, thereby preventing its leaching.

#### 5.1.3 Lead concentration results

• Unencapsulated modules show significant variation in lead concentration, with most samples exceeding the RCRA (resource conservation and recovery act) lead limit indicated by the red dashed line.
• Encapsulated, Unbroken modules generally demonstrate very low lead concentrations, well below the RCRA limit, highlighting the effectiveness of encapsulation in containing lead leaching.
• Encapsulated, Broken modules without a sequestration layer show a slight increase in lead concentration but are still below the RCRA limit. However, those with a sequestration layer have markedly lower lead concentrations, even when broken, underscoring the layer's additional protective effect.

The figure effectively demonstrates that encapsulation significantly reduces lead leaching from PSCs, particularly when unbroken. Additionally, the inclusion of a sequestration layer provides an extra level of protection, crucially maintaining lead concentrations below hazardous levels, even when the encapsulation is compromised. This data underscores the importance of both encapsulation and sequestration layers in managing environmental risks associated with the deployment of perovskite solar technologies.

**Fig. 8:**
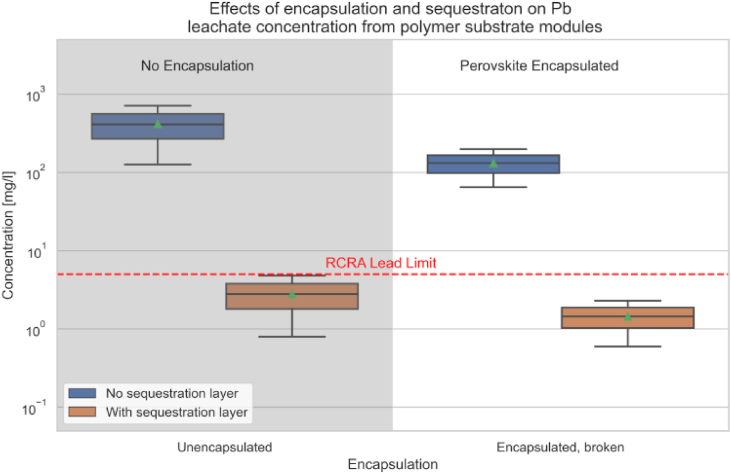
Leaching data of polymer substrate perovskite cells and modules with and without encapsulation [124].

Fig. 8 highlights the impact of encapsulation and sequestration layers on the Pb leachate concentrations from PSCs with polymer substrates. The figure shows that cells equipped with sequestration layers significantly reduce Pb concentrations, with measurements at 2.8 mg/L for unencapsulated and 1.45 mg/L for damaged encapsulated cells. In stark contrast, cells without sequestration layers exhibit much higher concentrations of 417 mg/L and 132 mg/L, respectively. This stark difference underscores the effectiveness of sequestration techniques in significantly mitigating lead contamination from both damaged and unencapsulated PSCs.

Furthermore, the figure demonstrates the crucial role of encapsulation in reducing lead leaching, where encapsulated cells, even when damaged, maintain lead concentrations far below the safety threshold. The inclusion of a sequestration layer acts as an additional protective barrier, enhancing safety measures and ensuring that lead concentrations remain minimal, thereby preventing environmental contamination. These findings emphasize the importance of implementing both encapsulation and sequestration layers in the design of perovskite solar modules to safeguard against lead leakage and ensure environmental safety.

### 5.2 Lead-free perovskites

Among the numerous Pb-free alternatives for PSCs, such as those utilizing Sn, germanium (Ge), bismuth (Bi), and double perovskites, Sn-based perovskites like CsSnI_3_, MASnI_3_, and FASnI_3_ emerge as leading candidates for crafting non-toxic active layers in solar cells. Initial research highlighted CsSnI_3_ perovskites' effectiveness as light absorbers across various solar cell types [125]. Building on this, the teams led by Snaith and Kanatzidis advanced Sn-based PSCs, achieving PCEs of 6.4 % and 5.73 %, respectively. This underscores Sn's potential as a practical Pb substitute, attributed to their analogous ionic radii and consequently similar optoelectronic and crystallographic characteristics [126]. Sn-based PSCs exhibit a direct bandgap within the optimal range of 1.2–1.4 eV, aligning well with the Shockley-Queisser limit for solar cell efficiency. The theoretical charge carrier mobility of Sn-based perovskites could surpass that of Pb-based ones due to Sn's smaller effective mass and reduced interaction with phonons. However, challenges such as energy level mismatches with charge transport materials, high susceptibility of Sn^2+^ to oxidation to Sn^4+^, and issues with film quality and stability due to rapid crystallization and high defect density have initially hindered their application.

Significant research efforts over the past decade have focused on addressing these challenges through various strategies, including engineering approaches to prevent Sn oxidation, control perovskite growth, and enhance charge extraction. By 2021, these efforts culminated in a record PCE of 14.81 % (certified at 14.03 %) for Sn-based PSCs [127]. Research continues to explore methods like using additives, modifying composition, optimizing device architecture, and developing 2D/3D heterostructures to overcome the remaining obstacles and fully realize the potential of Sn-based perovskites as a non-toxic alternative for solar cell applications.

### 5.3 Less-lead perovskite

While emerging as potential solutions to the concerns surrounding Pb-based PSCs, Pb-free alternatives often lack comparable efficiency and stability, hindering their practical application competitiveness. Sn–Pb PSCs, which blend Pb and Sn within the perovskite structure, offer a balanced approach by reducing toxicity while maintaining high-performance levels. However, challenges arise due to the differing crystallization rates of Sn and Pb in these perovskites, leading to uneven growth and defect formation that hampers electron flow. Recent research addressed the synchronizing of the crystallization processes and encouraging uniform crystal growth to improve film quality and device performance [128]. This has been shown to decrease internal stresses in the perovskite, enhancing the overall quality of the solar cells. Additionally, the combination of Pb and Sn adjusts the perovskite's bandgap to a more optimal range of 1.2–1.3 eV due to the bowing effect, which is lower than that observed in solely Pb - or Sn-based PSCs.

The first Sn–Pb PSCs were introduced in 2014 and achieved efficiencies of 4.18 % and 7.37 %, respectively [129]. Recent advancements have pushed efficiencies beyond 23 %, with the current highest recorded efficiency at 23.6 % [130]. Efforts to enhance the performance of Sn–Pb PSCs have included the use of additives to prevent oxidation, n-doping techniques, and the development of 2D perovskite structures on the surface.

### 5.4 Recycling: environmental and economic perspective

The recycling and recovery of toxic materials in M-PSCs are essential strategies for mitigating their environmental impact. Europe's Directive 2012/19/EU mandates producers to manage the collection, recycling, and recovery of electrical and electronic waste, aiming to reduce waste and promote resource efficiency [131]. PSCs feature both organic and inorganic elements. Recycling organic compounds, such as the costly Spiro-OMeTAD, is challenging due to their fragile structure and the toxic solvents required, like toluene, making them less suitable for large-scale industrial processes [132]. Table 3 shows the cost and quantity analysis of embedded elements in PSCs. An examination of the cost and quantity of embedded elements in PSCs serves as an essential foundation for understanding their economic viability and potential environmental challenges [133].

**Table 3:** PSCs embedded elements costs and amounts [133].

Thickness (μm)	Layer	Material	Cost (USD/m^2^)	Weight (g/m^2^)	Typical device types	References
**0.1**	Back contact	Au	110.77	1.9	n–i–p and p–i–n	[134]
		Ag	1.03	1.31	n–i–p and p–i–n	[135]
**5**–**10**	Carbon		<0.3	12.8	n–i–p or HTL-free mesoporous	[135,136]
**0.1**	Hole transport layer	Spiro-OMeTAD	9.28	0.13	n–i–p	[134,135]
		PEDOT.PSS	0.04	0.15	p–i–n	
**0.3**–**0.4**	Light harvesting layer	M-PSCs^a^	1	0.47–1.24^b^	All types	[134,135,137]
**0.025**–**0.25**	Electron transport layer	TiO_2_	0.06	0.53	n–i–p and HTL-free mesoporous	[43,135,136]
		ZnO	0.07	0.7	n–i–p	
		SnO_2_	0.02	0.87	n–i–p	
		PCBM	6.02	0.19	p–i–n	
**2000**	Conductive substrate	FTO/glass	100	5040	n–i–p and HTL-free mesoporous	[43,135,137]
**250**		PET/ITO	93.87	200	p–i–n	[138,139]

a The estimations for cost and weight are grounded in MAPbI_3**.**_

b A weight of 0.47 g/m^2^ is associated with a MAPbCl_3_ layer, having a density of 1.576 g/cm³, for a 300 nm layer thickness; meanwhile, a weight of 1.24 g/m^2^ is linked to a MAPbI_3_ layer with a density of 4.119 g/cm³, also for a thickness of 300 nm [137].

The cost of gold (Au) is determined based on a market rate of 58.3 USD/gram, for a density of 1.9 g/m^2^ in a layer of Au 100 nm thick. Similarly, the price of silver (Ag) is calculated with an Ag rate of 0.8685 USD/gram, for a density of 1.31 g/m^2^ in a 100 nm thick layer of Ag. The expense for Polyethylene terephthalate (PET) coated with conductive ITO is sourced from Thorlabs. Despite efforts to find alternatives, Spiro-OMeTAD (Table 3) remains critical for achieving top efficiency in state-of-the-art PSCs. Conversely, inorganic materials like TiO_2_, SnO_2_, and ZnO are cheaper and more abundant. The use of Pb in PSCs, deemed to have a negligible environmental impact by LCA, still lacks a comprehensive understanding of its market implications, emphasizing the need for effective recycling to mitigate environmental risks and driven by economic incentives [140]. Recent studies have explored in situ recycling of Pb iodide and a sequential recycling process for PSCs that allows for the recovery of the FTO-coated glass substrate [141]. However, the recovery and purification of solvents from these processes are less explored, and the flammability and toxicity of solvents like DMF pose significant industrial challenges [142]. This underscores the necessity for scaling up solvent-based recycling methods from the lab to industrial levels.

## 6 Conclusion and future outlook

### 6.1 Conclusion

This comprehensive review has shed light on the significant advancements and persistent challenges in the development of PSCs, focusing on M-PSCs and C–PSCs. Despite the potential of PSC technology as a high-efficiency, low-cost renewable energy solution, environmental concerns, especially regarding toxic Pb use and the stability and degradation issues of these solar cells, present considerable obstacles to their widespread adoption and sustainability. The exploration of Pb-free perovskites, innovative encapsulation techniques, and the recycling of PSC components emerge as pivotal strategies to mitigate the environmental impact of these technologies. Nonetheless, achieving a harmonious balance between technological advancements and environmental considerations necessitates continued research into sustainable and safe PSC technologies. Such research is crucial not only for reducing the environmental footprint of these promising energy sources but also for aligning with global renewable energy objectives and the overarching goal of sustainability.

The future of PSC technology appears promising but requires concerted efforts to address environmental and health concerns. Research should continue to develop non-toxic materials, improve encapsulation techniques, and enhance the recycling and reuse of PSC components. Innovations in device architecture, materials science, and engineering are crucial for advancing PSC efficiency, stability, and sustainability.

### 6.2 Recommendations

• ***Development of Lead-Free and Less-Lead Perovskites:*** Researchers should accelerate efforts to find efficient and stable alternatives to Pb in PSCs. The exploration of materials like Sn, Ge, and Bi, as well as double perovskite structures, may offer viable paths forward.
• ***Advanced Encapsulation Techniques:*** To mitigate environmental risks, developing robust encapsulation methods that can prevent the leakage of toxic materials from damaged or degraded PSCs is essential. Self-healing encapsulation materials could significantly reduce the environmental impact.
• ***Recycling and Reuse Strategies:*** Establishing effective recycling processes for PSC components is crucial for minimizing waste and recovering valuable materials. Research into solvent recovery and purification and the development of environmentally friendly manufacturing processes should be prioritized.
• ***Comprehensive Environmental Impact Assessments:*** Ongoing research should include holistic assessments of the environmental and health impacts of PSC technologies. This includes detailed studies on the bioavailability and toxicity of materials used in PSCs and the long-term effects of PSC deployment on ecosystems and human health.
• ***Policy and Regulatory Frameworks:*** Governments and regulatory bodies should develop and implement guidelines and standards that encourage the safe development, use, and disposal of PSC technologies. This includes regulations on the use of toxic materials, requirements for recycling and waste management, and incentives for adopting green manufacturing practices.
• ***Public and Private Sector Collaboration:*** Integrating academia, industry, and government is critical for advancing sustainable PSC technologies. Joint research initiatives, public-private partnerships, and investment in green technologies can accelerate developing and commercializing of environmentally friendly PSCs.

By addressing these recommendations, the research and development community can ensure that PSC technologies contribute to a sustainable energy future while minimizing their environmental footprint and ensuring public health and safety.

## Data availability statement

All data generated or analyzed during this study are included in this published article. No additional data are available.

## Ethics declaration statement

Review and/or approval by an ethics committee was not needed for this study because the manuscript does not include human or animal participation.

## CRediT authorship contribution statement

**Faycal Znidi:** Writing – review & editing, Writing – original draft, Visualization, Validation, Supervision, Resources, Methodology, Investigation, Funding acquisition, Formal analysis, Data curation, Conceptualization. **Mohamed Morsy:** Writing – original draft, Methodology, Funding acquisition, Formal analysis. **Md. Nizam Uddin:** Writing – original draft, Methodology, Formal analysis, Conceptualization.

## Declaration of competing interest

The authors declare that they have no known competing financial interests or personal relationships that could have appeared to influence the work reported in this paper.
